# PathoEye: A deep learning framework for the whole-slide image analysis of skin tissue

**DOI:** 10.1016/j.csbj.2025.11.052

**Published:** 2025-11-23

**Authors:** Yusen Lin, Feiyan Lin, Yongjun Zhang, Jiayu Wen, Guomin Li, Xinquan Zeng, Hang Sun, Hang Jiang, Jingxia Lin, Teng Yan, Ruzheng Xue, Hao Sun, Bin Yang, Jiajian Zhou

**Affiliations:** a Dermatology Hospital, Southern Medical University, Guangzhou, Guangdong, China; b Nanfang Hospital, The First School of Clinical Medicine, Southern Medical University, Guangzhou, Guangdong, China; c Warshel Institute for Computational Biology, The Chinese University of Hong Kong, Shenzhen, Guangdong, China; d School of Medicine, The Chinese University of Hong Kong, Shenzhen, Guangdong, China; e Department of Chemical Pathology, Li Ka Shing Institute of Health Sciences, The Chinese University of Hong Kong, Hong Kong, China

**Keywords:** Whole-slide images, Skin aging, Basement membrane zone, Deep learning, Radiomics

## Abstract

**Objective:**

To provide an interpretable computational framework for examining whole-slide images (WSI) in skin biopsies, PathoEye focuses on the dermis-epidermis junctional (DEJ) areas, also known as the basement membrane zone (BMZ), to enrich the pathological features of various skin conditions.

**Method:**

We presented PathoEye for WSI analysis in dermatology, which integrates epidermis-guided sampling, deep learning and radiomics. It enables the semantic segmentation of the BMZ automatically and extracts distinct features associated with various skin conditions.

**Results:**

PathoEye outperforms the existing methods in multi-class classification tasks involving various skin conditions by leveraging the BMZ-centric segmentation approach. It enables the investigation of histopathological aberrations in aged skin compared with young skin. Additionally, it highlighted the texture changes in the BMZ of young skin compared with aged skin. Further experimental analyses revealed that senescence cells were enriched in the BMZ, and the turnover of basement membrane (BM) components, including COL17A1, COL4A2, and ITGA6, was increased in aged skin.

**Conclusion:**

PathoEye is a WSI analysis tool that focuses on the features of the BMZ related to various skin conditions. The BMZ-centric patch sampling method improves the performance of the classification model for skin diseases.

## Graphical Abstract

**Figure:**
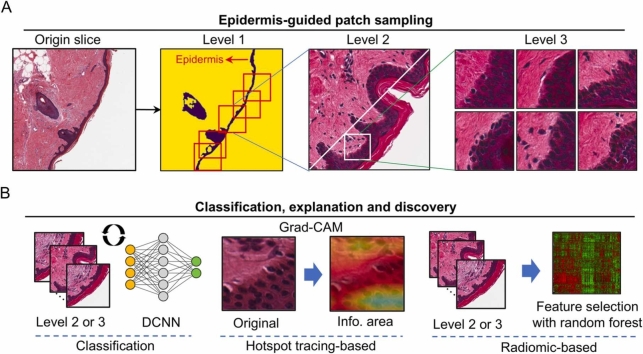
PathoEye is designed for whole slide imaging (WSI) analysis in dermatology by integrating epidermis-guided sampling (A), deep learning and radiomic analyses (B). It effectively identifies the distinct features of various skin conditions linked to the basement membrane zone (BMZ). PathoEye outperforms existing methods in both binary and multi-class classification tasks for healthy and diseased skin.

## 1 Introduction

Histological examination of skin biopsy slides is the gold standard for diagnosing most skin diseases in clinical settings [1], [2]. Advances in technology have enabled the accumulation and utilization of high-resolution whole-slide images (WSIs) in routine clinical practice [2], [3], [4], [5]. Currently, whole-slide imaging systems allow pathologists and researchers to visually inspect only a limited number of WSIs [6], [7], [8]. Nonetheless, summarizing features for comparative analysis across a large set of WSIs remains challenging: 1) precisely extracting the targeted areas of WSIs; 2) exploring novel features of histological images in an automatic manner [2], [9], [10], [11].

Recent studies have applied various algorithms and techniques to identify and quantify different features and patterns in the WSI of skin tissues [12], [13], [14], [15], [16], [17], [18], [19], [20], [21]. For example, Chen et al. developed a generalized and efficient model for the hematoxylin and eosin (H&E) stained WSI analysis, enabling a fast and scalable search of a large dataset by analyzing the down-sampling patches from the region of interest (ROI) [22], [23]. Similarly, Zheng et al. demonstrated that FastMDP-RL can automatically detect melanoma with WSIs using segmentation of ROI [22]. However, these methods only segment the tumor area and focus on the informative features derived from ROIs while losing information on other compartments. Interestingly, some studies have indicated that the epidermis and the basement membrane zone (BMZ) are suitable for WSI analysis, as numerous cytological features are observed in these areas [23], [24]. Notably, the basement membrane (BM) provides tissue integrity, elasticity, and mechanical signaling within the cell niche, and aberrations in the BMZ of various skin diseases have been observed [25], [26]. Therefore, we propose that patches sampled along the BMZ in WSIs could provide valuable insights for comparative analyses of different skin conditions.

Manual annotation of the WSI of skin tissue is labor-intensive and time-consuming. Most existing methods require substantial, manually curated WSIs, which limits their applications in clinical diagnosis [22], [23], [27]. Interestingly, radiomics involves the extraction of quantitative metrics from medical images, which can be easily retrieved at high speed and require fewer computational resources compared to traditional methods [28], [29], [30], [31]. On the other hand, the deep convolutional neural network (DCNN) and foundation model have been employed in WSI analysis by dividing the ROIs into several patches. However, these approaches struggle to summarize the features of a disease group, often resulting in less interpretable outcomes [13], [32], [33], [34]. Grad-CAM has been developed to visualize the informative features of DCNN models through gradient-based localization, thereby enhancing the interpretability of deep neural networks [35]. Therefore, a computational framework incorporating radiomic metrics and the interpretable Grad-CAM model may facilitate the exploration of novel features in large-scale WSI studies.

In this study, we developed PathoEye to automatically localize BMZ-centric patches in WSIs and extract distinct features associated with various skin conditions. We then examined the impact of our strategy on performance in multi-class classification tasks involving multiple skin conditions, comparing it with existing methods. Our work will highlight the usefulness of the BMZ-centric patches sampling in WSI analysis and draw attention to the functional role of BMZ integrity in skin aging.

## 2 Methods and materials

### 2.1 Data collection and sample acquisition

The WSI dataset comprises 511 exposed skin tissues and 406 non-sun-exposed skin tissues, which were downloaded from the GTEx project [36]. We defined the young group as those aged 20–39 years old (n = 83) and the aged group as those aged 60–79 years old (n = 83). Skin biopsies for Immunofluorescence (IF) and Immunohistochemistry (IHC) staining were obtained with ethical approval at the Dermatology Hospital of Southern Medical University (IRB#C0225020). The H&E WSIs of 4 skin diseases are retrieved from the Dermatology Hospital of Southern Medical University. All participants have signed an informed consent. The demographic information of all samples involved in this study is included in Table S1.

### 2.2 Epidermis extraction

**Fig. 1:**
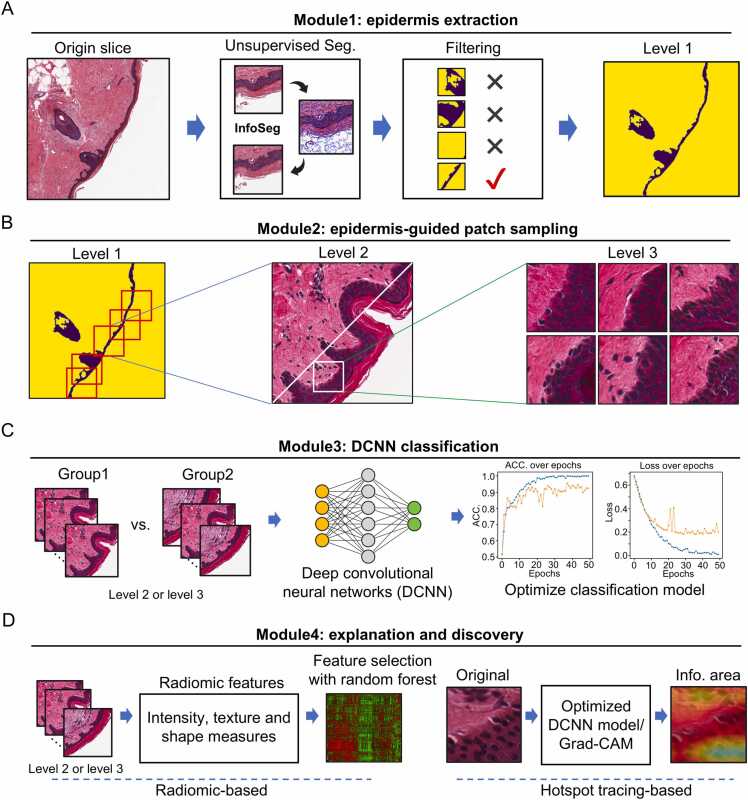
PathoEye: a deep learning framework for histopathological image analysis of skin tissue. A. the scheme for epidermis extraction in a histopathological image of skin tissue; B. three levels of segregated images used in PathoEye analyses; C. DCNN classification model with level 2 image as input. D. the explanation and discovery module for WSI analysis.

The WSIs are subjected to color normalization using Macenko et al.’s method [21], [37]. We adopted the InfoSeg [38] algorithm to establish the epidermis extraction module, which leverages the benefit of the automated segmentation algorithm and a customized image filter strategy (Fig. 1A). This process can be summarized in three parts: 1) Superpixel clustering. Pixels with similar patterns of epidermis were clustered as a superpixel using the Felzenszwalb algorithm [39]. These superpixels serve as a guide for optimizing the deep learning process. 2) Deep learning process. A chain of deep learning modules consists of a convolutional layer, a rectified linear unit (ReLU) activation layer, and a batch normalization layer. Then, the model employs a 1 × 1 convolutional layer followed by a Softmax layer for pixel classification, resulting in a mask representing the epidermis region after several iterations (Figure S1A); 3) Refinement. We removed the level 1 images containing numerous hair follicles, glands, and vascular structures if the mask did not span the diagonal of the image. Subsequently, we used a corrosion expansion algorithm to eliminate image flaws, resulting in a level 1 image with the epidermis oriented diagonally (Figure S2B). The image and the informative mask were subjected to downstream analyses.

### 2.3 Performance evaluation

**Fig. 2:**
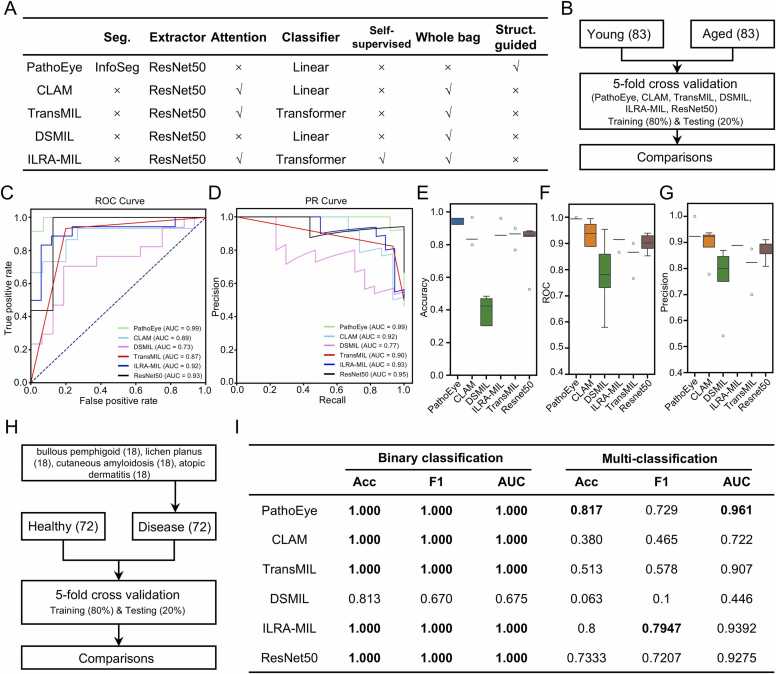
PathoEye outperforms the existing methods in classification tasks. A. The characteristics of PathoEye, CLAM, TransMIL, DSMIL and ILRA-MIL in the comparison analyses. B. The analysis scheme for the binary classification of young and aged skin tissues (n = 83 per group). C and D. The receiver operating characteristic (ROC) curve (C) and the precision-recall (PR) curve (D) showed that PathoEye has a better performance compared with CLAM, TransMIL, DSMIL, ILRA-MIL or ResNet50. E-G. the boxplots showed that PathoEye performs better than the existing methods in the 5-fold cross-validation analyses in terms of accuracy (E), AUC (F), and PR (G). H. the scheme of classifying the diseased and the healthy skin tissues (n = 72 per group). I. the comparative analyses of the performance of 6 models including PathoEye, CLAM, TransMIL, DSMIL and ILRA-MIL and ResNet50.

The level 2 patches sampled from the WSI images derived from the GTEx project and multiple skin diseases were used to evaluate the performance of PathoEye. ResNet50, CLAM, TransMIL, DSMIL, and ILRA-MIL were downloaded from GitHub and implemented under the same computational environment, following the official instructions. For the ablation experiment, the pure ResNet50 is included. We split the patches at the WSI level, then we conducted 5-fold cross-validation analyses (80 % for training model and 20 % for testing model) for all models across four classification tasks, including distinguishing aged skin tissues from the young group, differentiating diseased skin tissues from healthy ones, and classifying skin tissues under five conditions (Fig. 2B and H, Table S8). To evaluate the performance of the five methods, we assessed the accuracy, receiver operating characteristic (ROC) curves, F1 scores, and the area under the precision-recall (PR) curve (AUC). The evaluation method is applied for external validation and cross-institutional testing on the Rocío Del Amor et al. dataset [40].

### 2.4 Thickness and rete ridge score calculation

**Fig. 3:**
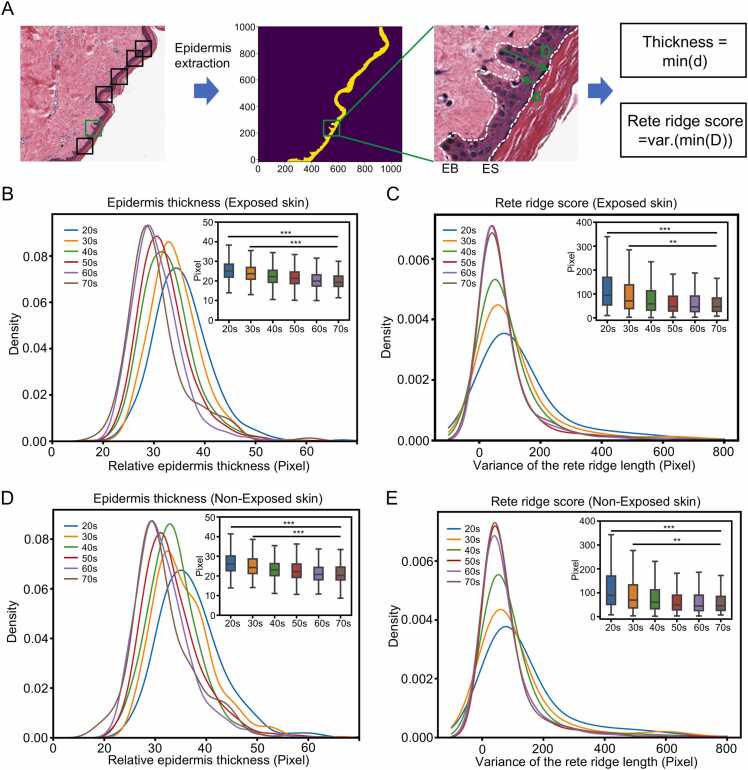
PathoEye shows a decrease in epidermal thickness and the variance of rete ridge length in aged skin. A. The schematic diagram illustrates the definition of the rete ridge score and thickness used to describe the morphology of the skin epidermis. B. the distribution of epidermic thickness in skin tissue of different age stages in sun-exposed condition; C. the changes of rete ridge length of BM in skin tissue of different age stages in sun-exposed condition; D. the distribution of epidermic thickness in skin tissue of different age stages in non-sun-exposed condition; E. the changes of rete ridge length of the BMZ in skin tissue of different ages in non-sun-exposed condition. These figures demonstrate that the thickness and rete ridge length of the epidermis decrease with skin aging. Pixel as the unit to measure the epidermis thickness and rete ridge length of the images collected with the same parameters. The sample sizes of different groups in the sun-exposed skin are listed as follows: 20 s, n = 43; 30 s, n = 40; 40 s, n = 83; 50 s, n = 171; 60 s, n = 156; 70 s, n = 19. The sample sizes of different groups in the non-sun-exposed skin are listed as follows: 20 s, n = 26; 30 s, n = 30; 40 s, n = 67; 50 s, n = 131; 60 s, n = 135; 70 s, n = 17. Statistical analysis was performed by two-tailed unpaired Student’s *t*-test; ns, not significant; *, *P* < 0.05; **, *P* < 0.01; ***, *P* < 0.001.

We defined EB and ES as the epidermis-dermis and epidermis-corneum boundary; The points along EB to ES or from ES to EB was used to quantitatively measure the rete ridge length and thickness, respectively (Fig. 3A and Figure S3). Then, the epidermis thickness as the mean of the shortest distance of a point from ESi to EB (${\mathrm{Distance}}_{{\mathrm{ES}}_{i},\mathrm{EB}},d$). The epidermis rete ridge is reduced along human skin aging, while the shortest distance from a point in the edge of basal layer (EBj) to the edge of stratum corneum (ES) (${\mathrm{Distance}}_{{\mathrm{EB}}_{j},\mathrm{ES}},D$) tends to remain constant in the epidermis. We then defined the epidermis rete ridge score as the variance of ${\mathrm{Distance}}_{{\mathrm{EB}}_{j},\mathrm{ES}},D$ represents how far a set of numbers is spread out from their average value. The higher the epidermis rete ridge score, the more rete ridge exists in the epidermis. The metrics were calculated as follows:

$$\mathrm{Thickness}=\mathrm{Mean}\{x|{\mathrm{Distance}}_{{\mathrm{ES}}_{i},\mathrm{EB}},0<i<m\}$$

$$\mathrm{Rete ridge score}=\mathrm{Variance}\{x|{\mathrm{Distance}}_{{\mathrm{EB}}_{j},\mathrm{ES}},0<j<n\}$$

### 2.5 Radiomic analysis

106 radiomic features were automatically extracted using the Pyradiomics package, utilizing level 2 or 3 patches as input [41]. The features were categorized into two groups: 1) first-order statistics, and 2) higher-order texture characteristics. First-order statistics comprise 18 basic metrics that reflect the symmetry, uniformity, and local intensity distribution of the measured voxels. In contrast, the higher-order texture features assess the heterogeneity differences within the epidermis areas in a texture format (Supplementary Notes). We then established a radiomic-based classifier using a random forest algorithm to classify young and aged skin images based on radiomic features. The informative features representing the differences between the two groups were obtained. We then ranked the features by the weight value, and the top 5 radiomic features were selected for further analysis.

### 2.6 DCNN-based hotspot tracing module

In the training process of DCNN classification, we employed a stochastic gradient descent (SGD) optimizer with a learning rate of 0.01 and a mini-batch size of 1, through a ResNet50 backbone pretrained on ImageNet. Specifically, a DCNN model is established for discriminating between young and aged skin using a pre-trained ResNet50 model as the backbone [42]. Then, we applied Grad-CAM [35] to trace the defects in the aged group using the trained model. The spots in the image associated with the aged skin were extracted using the derivative of the loss value and the activation value propagated from the trained model, resulting in a heatmap representing their activation values and the derivative of the loss value in the first convolutional layer (Figure S2). The red spot represents the importance of spatial features in terms of skin aging, while the blue spot represents less importance. All analyses are carried out at a computer node with Intel(R) Xeon(R) Gold 6230 CPU @ 2.10 GHz, 512 G RAM and NVIDIA A100 80 GB.

### 2.7 IF and IHC staining

For IF staining, the sections were prepared and incubated with rabbit anti-p16^INK4a^ antibody (1:100 dilution; Abcam, ab108349) and the Goat Anti-Rabbit secondary antibodies (1:500 dilution; Abcam, ab150078) as described previously [43]. Then, fluorescence images were obtained using a confocal (Nikon A1 +, Japan). For IHC staining, skin biopsies were fixed, sectioned and incubated with the antibodies described previously [44]. Then, the WSIs were obtained using NanoZoomer (Hamamatsu, Japan). Finally, we randomly selected 15 regions in the BMZ (ROI) of each IHC stain image for downstream quantification analyses, and the intensity was analyzed using ImageJ (version 1.54 f).

### 2.8 Statistical analysis

Data was analyzed using Python (version 3.7) and represented as mean ± standard deviation (S.D.). All tests were two-sided, and *P* < 0.05 was considered statistically significant.

## 3 Results

### 3.1 PathoEye: a deep learning framework for WSI analysis of skin tissue

The comparative analysis of large repositories of gigapixel WSIs requires intensive manual annotation and substantial computational resources, which limits their applications in dermatological research [45]. Here, we developed PathoEye to automatically analyze the anatomy and pathological features of WSIs using a BMZ-centric patches sampling strategy. PathoEye consists four modules (Fig. 1): 1) epidermis extraction module; it incorporates an unsupervised segmentation algorithm InfoSeg [38] to roughly retrieve the epidermic area and apply a customized method to remove hair follicle, gland and vascular structure, resulting in level 1 images (Fig. 1A and Figure S1A). 2) epidermis guided patch sampling module; it generates 512 × 512-pixel patches (term as level 2 images) along the mask representing the epidermis region in WSIs, which composite the dermis, BM, epidermis, and stratum corneum; in the meantime, 128 × 128-pixel patches (term as level 3 images) were extracted from the level 2 images along the mask representing epidermis; they represent the details information of BMZ (Fig. 1B and Figure S1B). 3) Deep Convolutional Neural Networks (DCNN) classification module; the level 2 and level 3 images are subjected to a DCNN-based model for training a model for category classification (Fig. 1C and Figure S2). 4) exploration and discovery utilities; it extracts the radiomic features of the level 2 and level 3 images using Pyradiomics [41], resulting in 106 features per image, then establishes an optimized machine learning model for classification (Fig. 1D, left panel and Figure S4); on the other hand, we developed a hotspot tracing module to trace the important hotspot area of the trained model using Grad-CAM [35] (Fig. 1D, right panel and Figure S2), which represents the informative features for discriminating abnormal and normal skin tissues. PathoEye has three unique features: 1) it segments the epidermis of the skin WSIs automatically, significantly reducing the labor-intensive annotation; 2) it incorporates epidermis extraction and a BMZ-centric patches sampling, which effectively extracts the informative features of the WSIs; 3) the exploration utilities enable the discovery of the informative features in a large-scale comparative analysis of WSI datasets.

### 3.2 PathoEye outperforms the existing methods in classification tasks

PathoEye incorporates an innovative epidermis-guided patch sampling strategy that utilizes the pathological alteration of the BMZ in both level 2 and 3 images, which helps discriminate between multiple skin conditions. To test this, we collected the H&E-stained WSI images of skin biopsies from the young (n = 83) and aged (n = 83) skin from the GTEx project [36] for comparison analysis. Then, we compared the performance of PathoEye with CLAM [46], transMIL [47], DSMIL [48], and ILRA-MIL [17] for the classification tasks, as all of them incorporate ResNet50 but extract features using different strategies (Fig. 2A). Firstly, we compared their performance in discriminating between aged and young skin in level 2 images (Fig. 2B). As a result, the receiver operating characteristic (ROC) curve and the precision-recall (PR) curve showed that PathoEye has better performance (ROC-AUC = 0.99, PR-AUC = 0.99) compared with CLAM (ROC-AUC = 0.89, PR-AUC = 0.92), TransMIL (ROC-AUC = 0.73, PR-AUC = 0.77), DSMIL (ROC-AUC = 0.87, PR-AUC = 0.90) and ILRA-MIL (ROC-AUC = 0.92, PR-AUC = 0.93), indicating that the patch sampling method with biological implication outperforms the method with attention algorithm or while bag selection strategies (Fig. 2C and D). The boxplot showed that PathoEye performed better than the existing methods in the 5-fold cross-validation analyses in terms of accuracy, ROC-AUC, and PR-AUC (Fig. 2E-G), suggesting the robustness of PathoEye in discriminating between young and aged skin using WSIs. Furthermore, we compared the performance of PathoEye with that of existing methods in the binary and multi-class classification of skin diseases, including bullous pemphigoid (n = 18), lichen planus (n = 18), cutaneous amyloidosis (n = 18), and atopic dermatitis (n = 18) (Fig. 2H). The results showed that PathoEye performed similarly to CLAM, TransMIL, and ILRA-MIL in the binary classification tasks, while achieving better performance in the multi-class classification tasks (Accuracy = 0.817, F1 = 0.729, and AUC = 0.961) (Fig. 2I). Furthermore, the ablation experiment demonstrated that ResNet50, without the BMZ-centric patches sampling strategy, exhibits a weaker performance in multi-class classification tasks compared with PathoEye (Accuracy = 0.7333, F1 = 0.7207, and AUC = 0.9275) (Fig. 2I). We also performed an additional validation on a cross-institutional dataset, PathoEye, which again outperformed the other methods and had significantly higher accuracy compared to ResNet50 (Figure S7). Therefore, the excellent performance of PathoEye may be attributed to: 1) the patches sampled along the BMZ contain pathogenic features of various skin diseases; 2) a patch composites the dermis, BM, epidermis, and stratum corneum, which makes a consistent dataset and eliminates the noise from other anatomical regions. In summary, our analyses demonstrated that the epidermis-guided patch sampling strategy enhances the performance of the DCNN classification model, and PathoEye outperforms existing methods in classification tasks.

### 3.3 PathoEye shows a decrease in epidermal thickness and the variance of rete ridge length in aged skin

Next, we sought to explore the features of H&E-stained WSIs of different ages using the exploration and discovery utilities in PathoEye. To this end, we collected a larger WSI dataset (n = 917) of skin tissues derived from various ages and sun exposure conditions. Then, we calculated the epidermis rete ridge score as the variance of the shortest distance between the point of the basal layer to the stratum corneum boundaries and the epidermis thickness as the mean distance between them (Fig. 3A and Figure S3). As a result, we observed that the thickness of the epidermis was significantly decreased in aged skin in both sun-exposed and non-sun-exposed groups (Fig. 3B and D, Table S2). Interestingly, we found that the epidermis thickness gradually decreased from 30 years old and remained stable from 60 years old (Fig. 3B and D). In addition, we observed that the variance of rete ridge length at the epidermis-dermis boundary is gradually decreased during aging in both sun-exposed and non-sun-exposed groups (Fig. 3C and E, Table S3). Notably, the decrease in variance of rete ridge length begins at 20 years old and remains constant at 50 years old, preceding the reduction in epidermis thickness. Our analysis revealed a striking decrease in the thickness and variance of rete ridge length in the epidermis during skin aging, consistent with previous studies [42], [43], which demonstrates the utility of PathoEye in exploring quantitative features of WSIs.

### 3.4 PathoEye identifies the age-associated patterns in the epidermal layer and the basement membrane zone

**Fig. 4:**
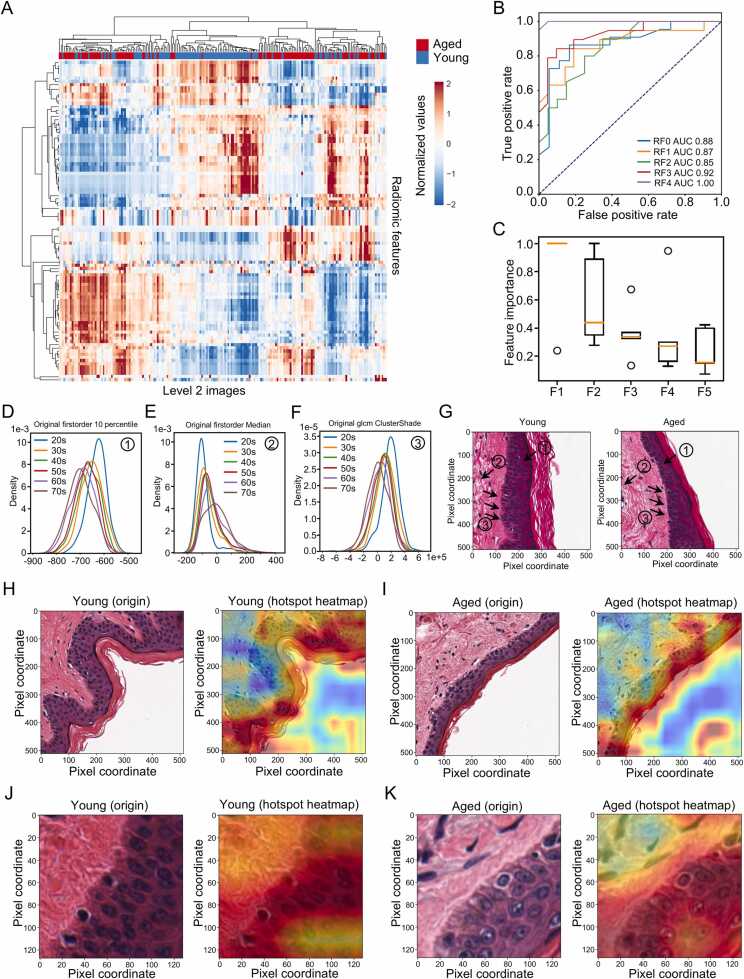
PathoEye identifies the age-associated patterns in the epidermal layer and the basement membrane zone. A. the heatmap clustering analysis of the radiomic features extracted from level 2 images, indicating that the radiomic features are distinct between young and old skin; B. Receiver Operating Characteristic (ROC) curve and Area Under Curve (AUC) analyses of Random Forest model for discriminating the young and aged skin; C. the top 5 crucial features in Random Forest model; F1: original first order 10 Percentile; F2: original first order Median; F3: original glcm Cluster Shade; F4: original first order Skewness; F5: diagnostics image-original Mean. D-F. Density plots show the distribution of the original first order 10 Percentile (D), original first order Median (E), and original GLCM Cluster Shade (F) with respect to skin aging. G. A snapshot illustrates the observed defects in the young and aged skin tissue. The labeled annotations correspond to the D, E, and F, respectively. H and I. the representative diagram showed the hotspot tracking of the level 2 image using Grad-CAM. J and K. the representative diagram showed the hotspot tracking of the level 3 image using Grad-CAM. The heatmap derived from Grad-CAM represents the importance of a specific region for the classification tasks; the red indicates the more importance of that region in the classification task, while the blue indicates less importance. Pixels are the unit of measure for the distance within the images because they were collected with the same parameters. The sample sizes of different groups in the sun-exposed skin are listed as follows: 20 s, n = 43; 30 s, n = 40; 40 s, n = 83; 50 s, n = 171; 60 s, n = 156; 70 s, n = 19.

Then, we applied PathoEye to explore the informative features associated with aged skin using level 2 images derived from young (20–29 years old) and aged (70–79 years old) skin. To avoid the influence of skin morphology, we only included the level 2 image containing BMZ (the dermis, epidermis, and epidermis diagonally across the patch). For the radiomic-based exploration, 101 radiomic features were subjected to clustering and prioritization analysis (Table S5). The top 5 features, ranked by weight value derived from the random forest (RF) algorithm, were then identified as the aged skin-associated features in level 2 and level 3 images (Fig. 4A and B, Figure S4 and S5). Notably, the three most significant features are the first-order 10th percentile, the first-order median, and the Gray-Level Co-occurrence Matrix (GLCM) cluster shade, which is associated with the saturation of color and texture complexity of an image (Fig. 4C). Expectedly, these features gradually increase or decrease with aging (Fig. 4D-F), and the changes are indeed observed in the epidermis (Fig. 4G). Specifically, the decrease in the first-order 10th percentile indicates a reduction in the number of epidermal cells in aged skin. In contrast, the increase of the first-order median indicates the fibrosis of the BMZ in the aged skin. The decrease in the Gray-Level Co-occurrence Matrix (GLCM) cluster shade suggests a disruption of collagen distribution and loss of cell type diversity in aged skin. For the hotspot tracing-based exploration, we traced the regions representing the differences between the young and aged skin using the established DCNN-based classification model (Figure S4, the bottom panel). As a result, we found that the epidermis region and BMZ are informative (the hotspot region) for discriminating between young skin and aged skin (Fig. 4H and I). Our further investigation showed that the chronic aging-related effects in BMZ of the aged skin using a similar analysis scheme (Fig. 4J and K, Figure S5A-C, Table S6 and Table S7), consistent with the fact that the aberrations of BMZ are critical for epidermal stem cells (EpiSCs) functional decline in aged skin [25], [49]. In summary, PathoEye is a powerful tool for exploring the representative changes of different skin conditions in a zoom-in manner.

### 3.5 Validation of the age-associated defects observed in the aged skin

**Fig. 5:**
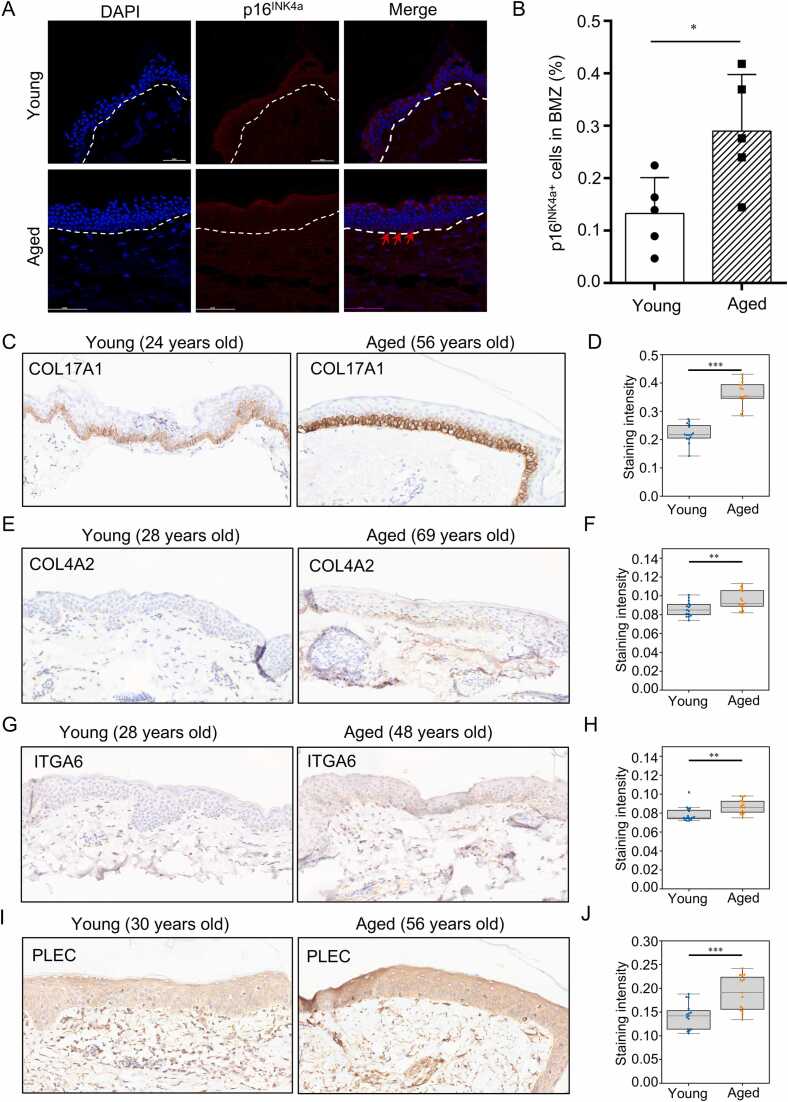
Validation of the age-associated defects in aged skin. A. IF staining reveals aged EpiSCs residing along the BM in aged skin (n = 5 per group). B. The boxplot illustrates the dysregulated expression levels of p16^INK4a^ in both young and aged skin, (n = 5 per group). C-J. IHC staining of COL17A1 (C and D), COL4A2 (E and F), ITGA6 (G and H) and PLEC (I and J) in the young and aged skin (n = 4 per group). Statistical analysis was performed by two-tailed unpaired Student’s *t*-test; ns, not significant; *, *P* < 0.05; **, *P* < 0.01; ***, *P* < 0.001.

Next, we plan to experimentally validate the observed age-related defects. Our immunofluorescence (IF) staining revealed that EpiSCs located in the basement membrane (BM) have a higher expression of p16^INK4a^ in aged skin compared to young skin, indicating a higher percentage of senescent EpiSCs in aged skin (Fig. 5A and B). Next, we investigated the protein expression of key components and regulators associated with the homeostasis of the BM and attached stem cells, including COL17A1, COL4A2, ITGA6, and PLEC [50], [51], [52], [53]. As expected, our immunohistochemistry (IHC) experiments revealed elevated levels of COL17A1, COL4A2, ITGA6, and PLEC proteins in aged skin compared to young skin (see Fig. 5C-J and Figure S6), indicating a transition in skin stiffness from young to aged. Notably, these changes may vary due to different environmental exposures or sampling site variability; therefore, the level of those markers should be tested in a larger sample size in the future. In summary, our experimental findings confirm that more senescent EpiSCs reside in the BM of aged skin, along with defects in the membrane zone (BMZ) components, thereby underscoring the potential application of PathoEye in dermatological research.

## 4 Discussions

In this study, we demonstrated that PathoEye is an interpretable histological image analysis pipeline capable of extracting and exploring novel features of the skin WSIs. It employs an automatic epidermis extraction method to reduce the need for labor-intensive manual annotation and a BMZ-centric patch sampling technique to enrich the pathogenic features of skin tissue, which enhances its performance in classification tasks.

The high resolution of WSIs and the complex morphological layers of skin tissues pose significant challenges in the comparative analysis of the large-scale dataset [27], [54]. Numerous studies have demonstrated the potential of deep learning algorithms in distinguishing tumor skin from the normal one [27], [33], [54], [55]. The target of deep learning frameworks is efficiently prioritizing the informative features associated with different groups. While CLAM, TransMIL, and DSMIL prioritize the important features of all sampled patches (including noisy patches) related to various groups, ILRA-MIL reduces the dimension of features and removes redundant patches by incorporating the Low-Rank Constraint (LRC) to improve classification performance. Interestingly, although the epidermis and BMZ provide most of the cytological features of various skin conditions [50], there is no epidermis/BMZ-focused method or classification model for skin WSI analysis to date. PathoEye automatically extracts the epidermis layer and applies a BMZ-centric patch sampling method to analyze the WSIs through a hierarchical strategy. It generates a limited number of anatomically consistent images that contain the dermis, epidermis, and BMZ along the same diagonal line, which helps radiomic feature extraction, category classification, and hotspot tracing in downstream analyses.

Previous reports have shown that many deep-learning models have achieved high accuracy in predicting melanoma or basal cell carcinoma [22], [33], [54]. In practice, these models output the prediction label with WSI as input without any explainable evidence corresponding to their decisions [32]. Here, PathoEye employs a DCNN-based hotspot tracing model and a radiomic analysis module to prioritize the evidence supporting the classification decisions. On the one hand, it incorporates a hotspot tracing module to trace the important hotspot area of the trained model using Grad-CAM algorithm, which provide intuitive and spatial visualization of the cytological features in skin biopsies; on the other hand, the radiomic features represent the global texture of the BMZ-centric patches, which represent the pathological aberrations of different layers in skin tissue, such as fibrosis, stiffness and alteration of cell count. With these advantages, our strategy enhances the application of computational pathology in diagnosing skin diseases and serves as a representative model for WSI analysis in dermatology.

Our current study has several limitations: 1) PathoEye extracts the informative region of WSIs using an epidermis-guided patch sampling strategy, eliminating the information from the other areas; this may somewhat weaken the predictive power. 2) We only applied PathoEye to discover the known and novel features by comparative analysis of WSIs derived from the young and the aged skin tissues, and several skin diseases. Further, a wide range of WSIs derived from multiple centers and a federated learning framework should be applied to evaluate the generalization of PathoEye [56].

## Ethical statement

Skin biopsies, both from diseased and normal tissue, for Immunofluorescence (IF), Immunohistochemistry (IHC), and Hematoxylin and Eosin (H&E) staining were collected from patients and donors who provided their consent at the Dermatology Hospital of Southern Medical University (SMUDH). The use of these skin biopsies has been approved by the SMUDH Institutional Review Board and Ethics Committee (IRB# C0225020).

## Code and data availability

The source code and a tutorial are available at GitHub (https://github.com/lysovosyl/PathoEye). The data is available at Figshare (https://doi.org/10.6084/m9.figshare.30566108.v1). CLAM, TransMIL, DSMIL ILRA-MIL and ResNet50 were downloaded from GitHub.

## CRediT authorship contribution statement

**Yusen Lin**: Writing – original draft, Software, Methodology, Investigation, Formal analysis, Conceptualization. **Feiyan Lin**: Writing – original draft, Visualization, Resources, Investigation, Formal analysis, Data curation. **Yongjun Zhang**: Writing – original draft, Methodology, Investigation, Formal analysis, Data curation. **Jiayu Wen**: Resources, Methodology, Data curation. **Li Guoming**: Resources, Data curation. **Xinquan Zeng**: Project administration, Methodology. **Hang Sun**: Project administration, Data curation. **Hang Jiang**: Methodology, Data curation. **Jingxia Lin**: Writing – review & editing, Project administration, Funding acquisition, Data curation. **Teng Yan**: Methodology. **Ruzheng Xue**: Validation, Supervision, Resources. **Hao Sun**: Supervision, Methodology. **Bin Yang**: Writing – review & editing, Validation, Supervision, Resources, Investigation, Conceptualization. **Jiajian Zhou**: Writing – review & editing, Validation, Supervision, Resources, Investigation, Funding acquisition, Conceptualization.

## Funding

10.13039/501100001809National Natural Science Foundation of China (NSFC) (32070792 to Z.J.); the Basic research project of Dermatology Hospital, Southern Medical University (BR202411 to J.X.); and Guangdong Province International and Hong Kong Macao Taiwan High Talent Exchange Special Project (109164881053 to Z.J.).

## Declaration of Competing Interest

The authors declare that they have no known competing financial interests or personal relationships that could have appeared to influence the work reported in this paper.
