# Supplementary material for: PathoEye: A deep learning framework for the whole-slide image analysis of skin tissue
Source: Comput Struct Biotechnol J. 2025 Nov 23;27:5391–400. doi: 10.1016/j.csbj.2025.11.052 (PMC12699262; doi:10.1016/j.csbj.2025.11.052)
Supplement: Supplementary file 1 — Supplementary material [file mmc1.pdf]

Supplementary Figures Summary

Figure S1. The architecture of epidermis extraction (InfoSeg model) and patch extraction with epidermis running diagonally, related to Figure 1. It provides the details of the epidermis extraction module and the customized filtering processes.

Figure S2. The architecture of DCNN for classification tasks, related to Figure 2 and 4. It provides the data processing steps for the DCNN classification, as well as Grad-CAM analyses, for performance evaluation purposes.

Figure S3. The scheme for calculating the thickness and rete ridge length of the epidermis, related to Figure 3.

Figure S4. The flowchart of WSI analysis in skin tissues of different age ranges, related to Figure 4. It provides the details of radiomic feature analysis in Figure 4.

Figure S5. PathoEye identifies defects in the basement membrane zone of aged skin, supported Figure 4. It provides supplemental results in level 3 images to support the defects found in the BMZ of the aged skin.

Figure S6. The expression level of four extracellular genes in young and aged human skin, supported Figure 5. The IHC staining images of the other three replicates are shown.

Figure S7. Validation of PathoEye performance using external datasets, related to Figure 2. It showed the performance of PathoEye using the Rocío Del Amor et al. dataset.

Figure S1.

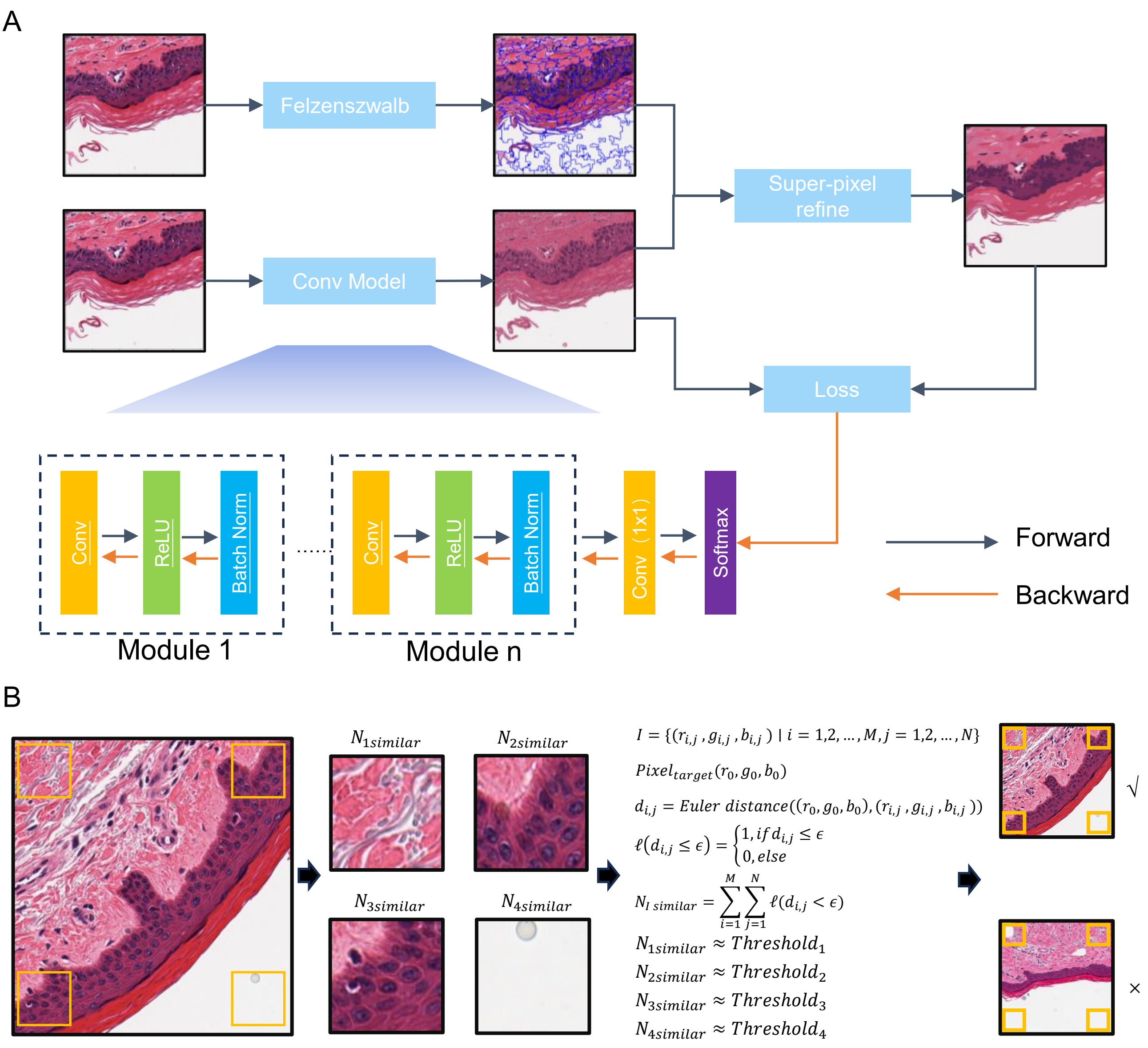

Figure S1. The architecture of epidermis extraction (infoSeg model) and patch extraction with epidermis running diagonally, related to Figure 1. A. it comprises a series of modules, including convolutional, ReLU, and batch normalization layers, including a 1x1 convolution layer and a softmax layer for pixel classification. Initially, an image is processed using the Felzenswalb algorithm to generate super-pixels. Then, the image is fed into the model to produce a mask, which is refined based on the super-pixels, resulting in a new mask. The new mask will optimize the current model, enhancing its ability to generate improved masks in subsequent iterations. This process is repeated several times for each image until saturation. B. The scheme for extracting patches with epidermis running diagonally. Four small patches were sampled from each point of the diagonal lines. For each patch, the similarity among patches was evaluated using *Euclidean distance*. Briefly, a higher similarity indicates the same pattern between two patches. Subsequently, the level 2 or level 3 images with epidermis oriented diagonally were identified if $N_{3similar} \approx N_{2similar}$ and $N_{1similar}$ distinct from $N_{4similar}$.

Figure S2.

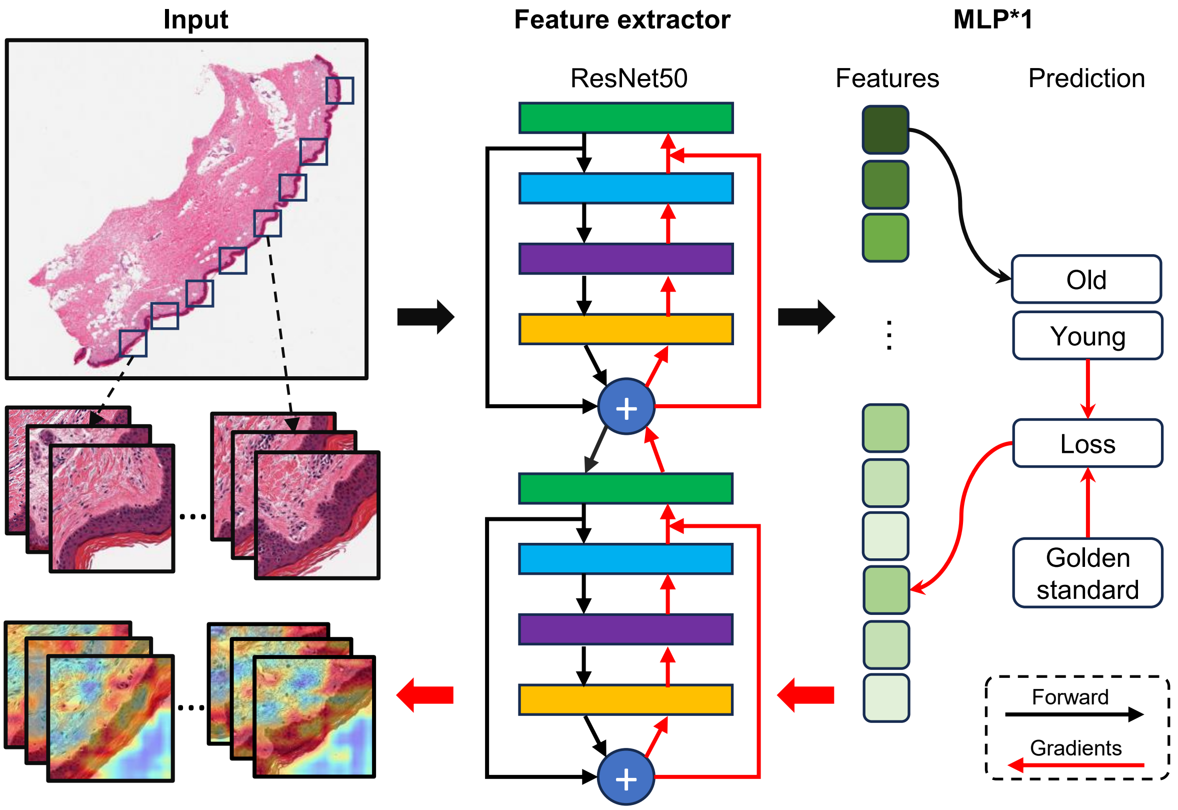

Figure S2. The architecture of DCNN for classification tasks, related to Figure 2 and Figure 4. The DCNN model was trained to discriminate between young and aged skin using the level 2 or level 3 images as input. Then, we traced the weighted aggregation of the feature maps and gradients in DCNN using the Grad-CAM algorithm to highlight the hotspot regions that were informative for prediction with the loss value, ground truth (golden standard), and prediction results.

Figure S3.

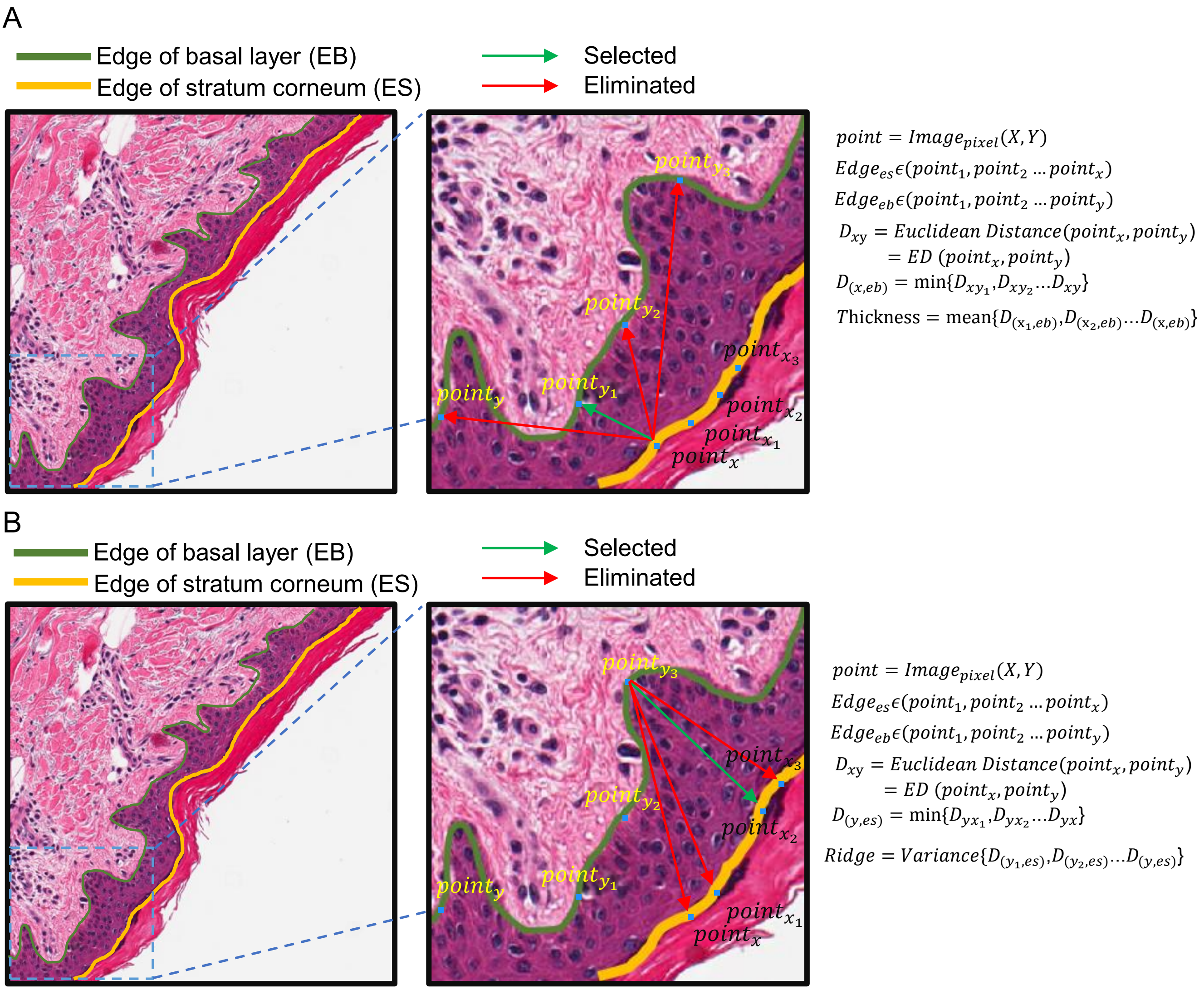

Figure S3. The scheme for calculating the thickness and rete ridge length of the epidermis, related to Figure 3. A. The illustration represents the formula and calculation method for the thickness of the epidermis. We defined the mean of the shortest distance between piontx and pointy as the thickness of the epidermis. B. The illustration represents the formula and calculation method for the rete ridge length variation of epidermis. We defined the rete ridge length as the distance from the points of the basal layer to the edge of the stratum corneum. Therefore, the variation of the rete ridge length along the edge of the basal layer represents the degree of fold in the epidermis.

Figure S4.

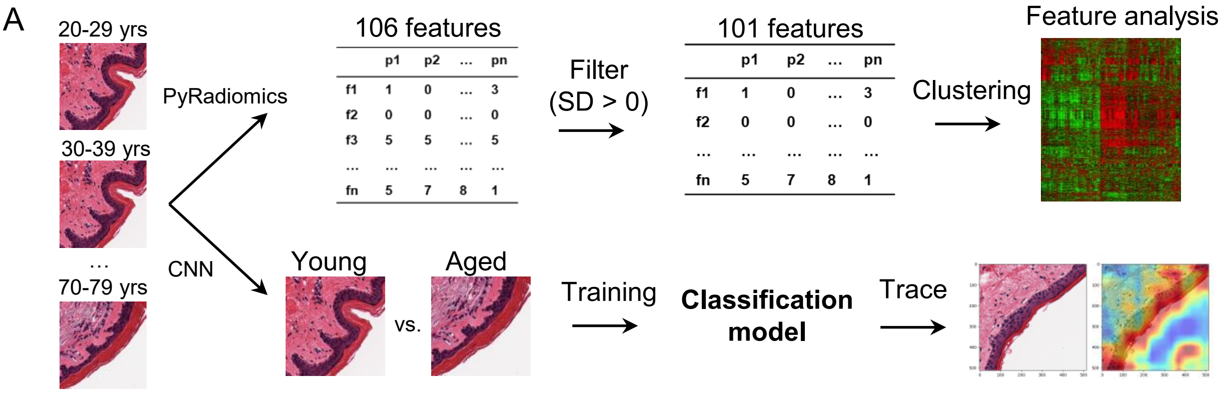

Figure S4. The flowchart of WSI analysis in skin tissues of different age ranges, related to Figure 4. The automated segmented images of varying age ranges were subjected to 1) radiomic feature analysis using PyRadiomics (top panel) and 2) classification analysis using CNN models (bottom panel).

Figure S5.

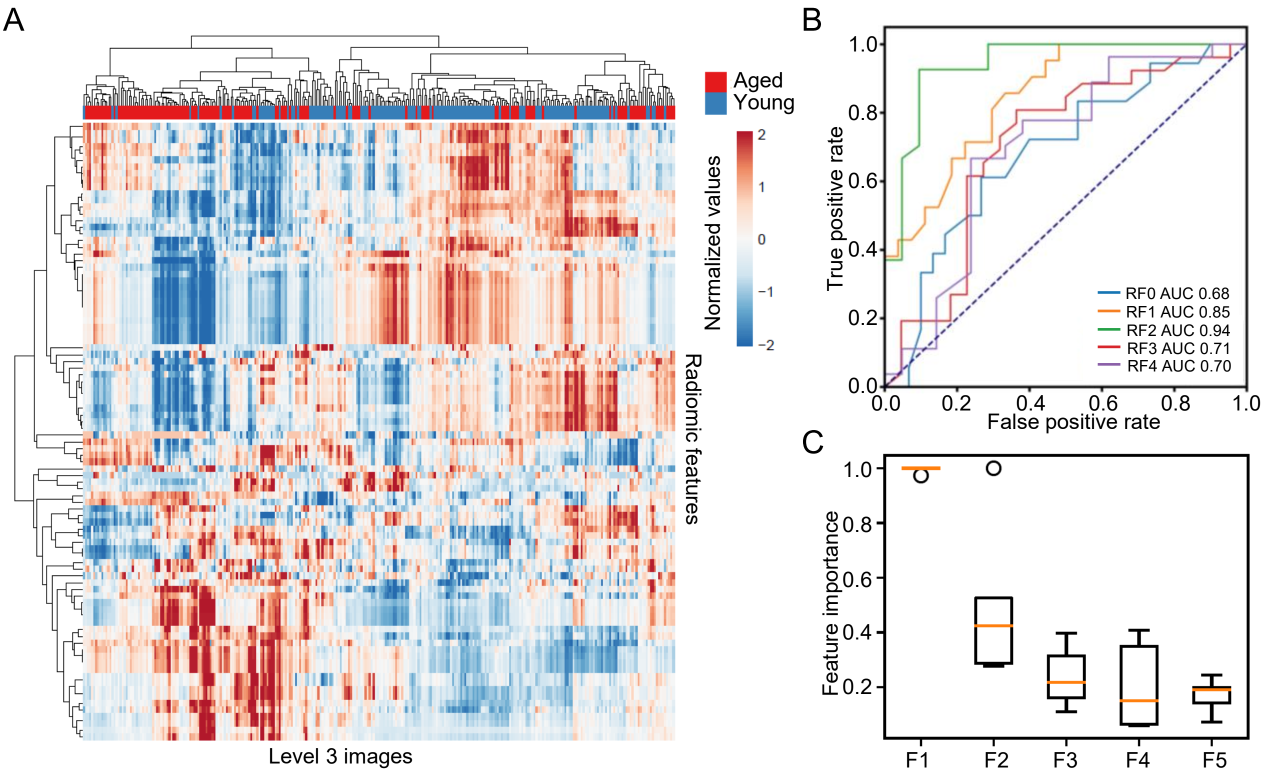

Figure S5. PathoEye identifies defects in the basement membrane zone of aged skin, supported Figure 4. A. the heatmap cluster analysis of the radiomic features extracted from the level 3 images; B. Receiver Operating Characteristic (ROC) curve and Area Under Curve (AUC) analyses of the Random Forest model for discriminating the young and aged skin; C. the top 5 important features from the Random Forest model; F1: original first order 10 Percentile; F2: original first order Median; F3: original glszm GrayLevelVariance; F4: original glcm DifferenceVariance; F5: original glcm MCC.

Figure S6.

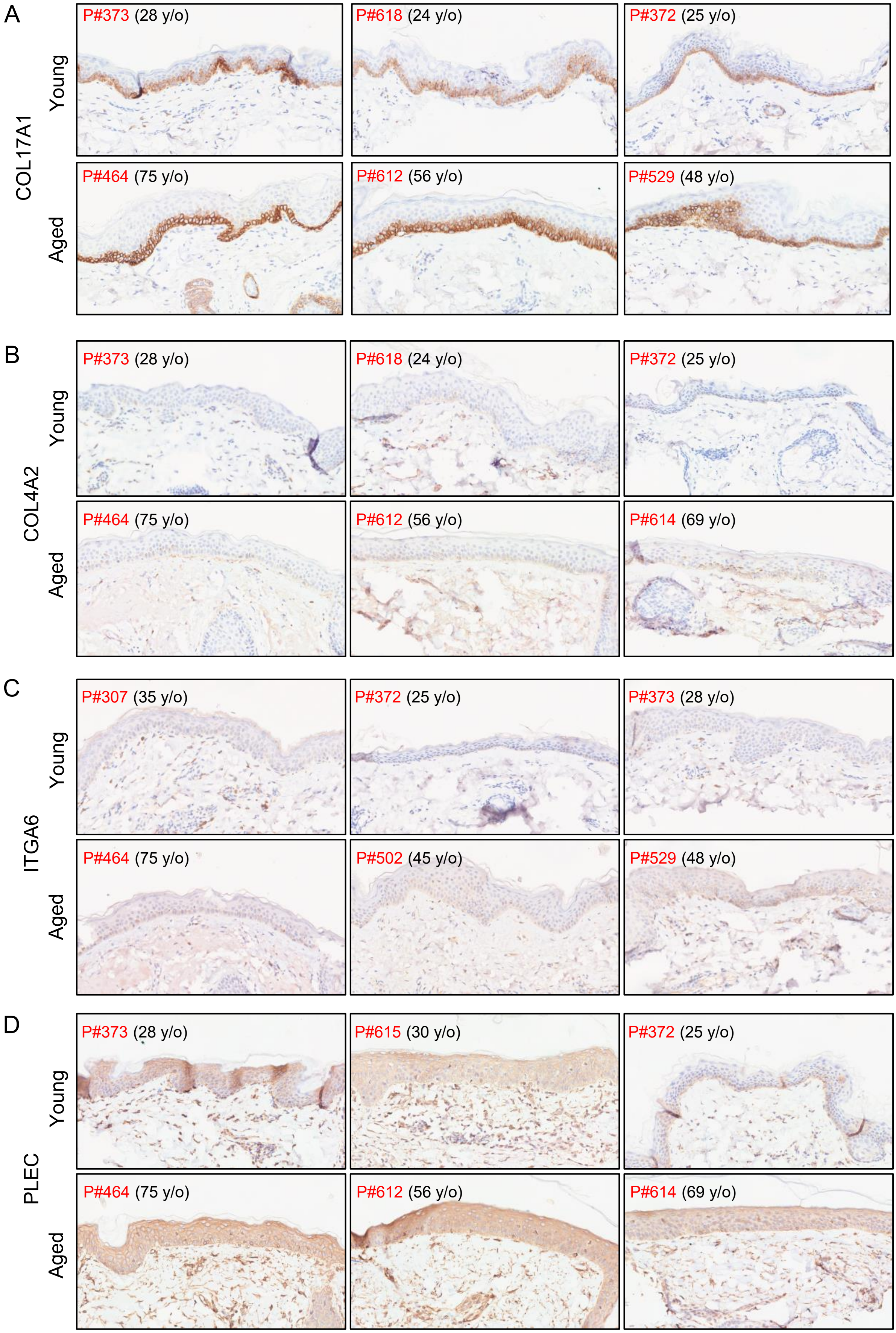

Figure S6. The expression level of four extracellular genes in young and aged human skin, supported Figure 5. Immunohistochemistry (IHC) staining for COL17A1 (A), COL4A2 (B), ITGA6 (C) and PLEC (D).

Figure S7.

A

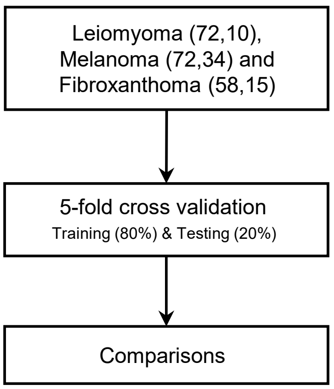

B

	Multi-classification		
	**Acc**	**F1**	**AUC**
PathoEye	**0.90446**	**0.89982**	0.97206
CLAM	0.88842	0.88724	0.97814
TransMIL	0.88076	0.87944	**0.98654**
DSMIL	0.42308	0.2136	0.664
ILRA-MIL	0.88842	0.88488	0.96846
ResNet50	0.86814	0.8564	0.96922

C

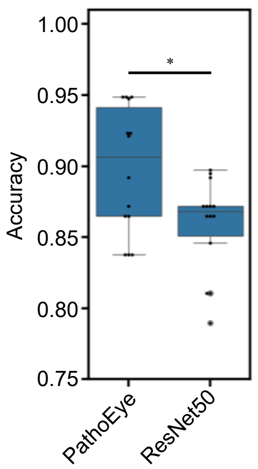

Figure S7. Validation of PathoEye performance using external datasets, related to Figure 2. A. The scheme for evaluating the performance of PathoEye using the Rocío Del Amor et al. dataset derived from two centers. B. the comparative analyses of the performance of 6 models, including PathoEye, CLAM, TransMIL, DSMIL, ILRA-MIL, and ResNet50. C. PathoEye has a significantly higher accuracy compared with RestNet50. Statistical analysis was performed by two-tailed unpaired Student’s t-test; ns, not significant; *, P<0.05; **, P<0.01.
