# Supplementary material for: PathoEye: A deep learning framework for the whole-slide image analysis of skin tissue
Source: Comput Struct Biotechnol J. 2025 Nov 23;27:5391–400. doi: 10.1016/j.csbj.2025.11.052 (PMC12699262; doi:10.1016/j.csbj.2025.11.052)
Supplement: Supplementary file 2 — Supplementary material [file mmc2.zip › Supplementary Notes.docx]

Supplementary Notes

Epidermis-guided patch sampling

A customized pipeline was established to simulate the zoom-in and anatomical inspection process in WSI analysis (Figure 1B). Briefly, it generates 512 x 512-pixel patches (term as level 2 images) along the mask representing the epidermis region in WSIs, which composite with the dermis, BM, epidermis, and stratum corneum; then, 128 x 128-pixel patches (term as level 3 images) were extracted from the level 2 images along the mask representing epidermis; they represent the detailed information of BMZ. Finally, the level 2 and 3 images are retained for downstream analyses using a customized filtering method to ensure the BM or epidermis diagonally runs across the patch (Figure 1B and Figure S1B).

**Radiomic features calculation**

For each WSI of skin tissue, we calculated 106 features using Pyradiomics package, resulting in 18 First Order features, 24 Gray Level Co-occurrence Matrix features, 16 Gray Level Size Zone Matrix features, 16 Gray Level Run Length Matrix features, 5 Neighbouring Gray Tone Difference Matrix features, 14 Gray Level Dependence Matrix features and 13 diagnostics. The calculation of those features uses the equation listed as follows:

**First Order Features**

1. **Energy**
2. **Total Energy**
3. **Entropy**

is a arbitrarily small positive number

1. **Minimum**
2. 10th percentile

The 10th percentile of X

1. 90th percentile

The 90th percentile of X

1. Maximum
2. Mean
3. Median

The median gray level intensity within the ROI.

1. Interquartile Range
2. Range
3. Mean Absolute Deviation (MAD)
4. Robust Mean Absolute Deviation (rMAD)
5. Root Mean Squared (RMS)
6. Skewness
7. Kurtosis
8. Variance
9. Uniformity

**Gray Level Co-occurrence Matrix (GLCM) Features**

1. **Autocorrelation**
2. Joint Average
3. Cluster Prominence
4. Cluster Shade
5. Cluster Tendency
6. Contrast
7. Correlation
8. Difference Average
9. Difference Entropy
10. Difference Variance
11. Joint Energy
12. Joint Entropy
13. Informational Measure of Correlation (IMC) 1
14. Informational Measure of Correlation (IMC) 2
15. Inverse Difference Moment (IDM)
16. Maximal Correlation Coefficient (MCC)
17. Inverse Difference Moment Normalized (IDMN)
18. Inverse Difference (ID)
19. Inverse Difference Normalized (IDN)
20. Inverse Variance
21. Maximum Probability
22. Sum Average
23. Sum Entropy
24. Sum of Squares

**Gray Level Size Zone Matrix (GLSZM) Features**

1. Small Area Emphasis (SAE)
2. Large Area Emphasis (LAE)
3. Gray Level Non-Uniformity (GLN)
4. Gray Level Non-Uniformity Normalized (GLNN)
5. Size-Zone Non-Uniformity (SZN)
6. Size-Zone Non-Uniformity Normalized (SZNN)
7. Zone Percentage (ZP)
8. Gray Level Variance (GLV)
9. Zone Variance (ZV)
10. Zone Entropy (ZE)
11. Low Gray Level Zone Emphasis (LGLZE)
12. High Gray Level Zone Emphasis (HGLZE)
13. Small Area Low Gray Level Emphasis (SALGLE)
14. Small Area High Gray Level Emphasis (SAHGLE)
15. Large Area Low Gray Level Emphasis (LALGLE)
16. Large Area High Gray Level Emphasis (LAHGLE)

**Gray Level Run Length Matrix (GLRLM) Features**

1. Short Run Emphasis (SRE)
2. Long Run Emphasis (LRE)
3. Gray Level Non-Uniformity (GLN)
4. Gray Level Non-Uniformity Normalized (GLNN)
5. Run Length Non-Uniformity (RLN)
6. Run Length Non-Uniformity Normalized (RLNN)
7. Run Percentage (RP)
8. Gray Level Variance (GLV)
9. Run Variance (RV)
10. Run Entropy (RE)
11. Low Gray Level Run Emphasis (LGLRE)
12. High Gray Level Run Emphasis (HGLRE)
13. Short Run Low Gray Level Emphasis (SRLGLE)
14. Short Run High Gray Level Emphasis (SRHGLE)
15. Long Run Low Gray Level Emphasis (LRLGLE)
16. Long Run High Gray Level Emphasis (LRHGLE)

**Neighboring Gray Tone Difference Matrix (NGTDM) Features**

1. Coarseness
2. Contrast
3. Busyness
4. Complexity
5. Strength

**Gray Level Dependence Matrix (GLDM) Features**

1. Small Dependence Emphasis (SDE)
2. Large Dependence Emphasis (LDE)
3. Gray Level Non-Uniformity (GLN)
4. Dependence Non-Uniformity (DN)
5. Dependence Non-Uniformity Normalized (DNN)
6. Gray Level Variance (GLV)
7. Dependence Variance (DV)
8. Dependence Entropy (DE)
9. Low Gray Level Emphasis (LGLE)
10. High Gray Level Emphasis (HGLE)
11. Small Dependence Low Gray Level Emphasis (SDLGLE)
12. Small Dependence High Gray Level Emphasis (SDHGLE)
13. Large Dependence Low Gray Level Emphasis (LDLGLE)
14. Large Dependence High Gray Level Emphasis (LDHGLE)
